# Association of *INSIG2* Polymorphism with Overweight and LDL in Children

**DOI:** 10.1371/journal.pone.0116340

**Published:** 2015-01-21

**Authors:** Anne-Marie Kaulfers, Ranjan Deka, Lawrence Dolan, Lisa J. Martin

**Affiliations:** 1 Division of Pediatric Endocrinology, University of South Alabama, Mobile, Alabama, United States of America; 2 Department of Environmental Health, University of Cincinnati School of Medicine, Cincinnati, Ohio, United States of America; 3 Division of Endocrinology, Cincinnati Children’s Hospital Medical Center and University of Cincinnati School of Medicine, Cincinnati, Ohio, United States of America; 4 Divisions of Biostatistics and Epidemiology, Division of Human Genetics, Cincinnati Children’s Hospital Medical Center and University of Cincinnati School of Medicine, Cincinnati, Ohio, United States of America; Children’s National Medical Center, Washington, UNITED STATES

## Abstract

**Background:**

Dyslipidemia and overweight are common issues in children. Identifying genetic markers of risk could lead to targeted interventions. A polymorphism of SNP rs7566605 near insulin-induced gene 2 (*INSIG2*) has been identified as a strong candidate gene for obesity, through its feedback control of lipid synthesis.

**Objective:**

To identify polymorphisms in *INSIG2* which are associated with overweight (BMI ≥ 85% for age) and dyslipidemia in children. Hypothesis: The C allele of rs7566605 would be significantly associated with BMI and LDL.

**Design/Methods:**

We genotyped 15 SNPs in/near *INSIG2* in 1,058 healthy children (53% non-Hispanic white (NHW), 37% overweight) participating in a school based study. Genotype was compared with BMI and lipid markers, adjusting for age, gender, and puberty.

**Results:**

We found a significant association between the SNP rs12464355 and LDL in NHW children, p < 0.001. The G allele is protective (lower LDL). A different SNP was associated with overweight in NHW: rs17047757. SNP rs7566605 was not associated with overweight or lipid levels.

**Conclusions:**

We identified novel genetic associations between *INSIG2* and both overweight and LDL in NHW children. Polymorphisms in *INSIG2* may be important in the development of obesity through its effects on lipid regulation.

## Introduction

Childhood obesity is associated with significant morbidity[1] and results in premature mortality[2]. The frequency of pediatric overweight, or body mass index (BMI) ≥ 85^th^ percentile for age[3], is now at 32%, making it a major public health concern. Despite studies demonstrating that genetics plays a significant role in obesity (heritability estimates of 30–70%) [4], there are still many questions regarding which genes contribute to obesity. Understanding the genetics of obesity is a key step in establishing mechanisms for the development of obesity and targeted strategies for primary and secondary prevention of overweight and obesity in children.

Due to the ability of the Insulin-induced gene 2 (*INSIG2*) to regulate adipogenesis and lipid storage [5], *INSIG2* is a strong candidate gene for obesity. *INSIG2* is involved in feedback control of lipid synthesis. When sterols are present in the cell, *INSIG2* blocks further cholesterol synthesis [6]. Engelking et al [7] showed that *INSIG2* knockout mice weighed more than controls and the mice had a higher accumulation of cholesterol and triglycerides in the liver. Krapivner et al [8] showed that *INSIG2* is also expressed in adipocytes, and this expression is enhanced during adipocyte regulation. These authors postulate that changes in adipocyte metabolism, due to functional polymorphisms in the *INSIG2* gene, can lead to changes in BMI.

Several studies have shown a significant association between *INSIG2* variant rs7566605 and obesity or BMI [4,9,10,11,12]. However, many other studies have failed to show association between rs7566605 and obesity phenotypes [8,13–29]. As obesity often co-occurs with dyslipidemia, other studies have examined the association with rs7566605 and one or more lipid markers (cholesterol, low-density lipoproteins (LDL), triglycerides, and/or high-density lipoproteins (HDL), with conflicting results [12,13,14,22,24,26,27,30,31]. This suggests that rs7566605 is not the causal variant; rather, another genetic variation in this region may be playing a role in development of obesity and/or dyslipidemia. However, few studies have examined genetic variation in the *INSIG2* gene more thoroughly, except genome wide association studies which due to the large sample sizes require large heterogeneous cohorts[4]. Given the heterogeneity of the results thus far, it is important to thoroughly study *INSIG2* genetic variation in homogenous cohorts.

We had the unique opportunity to analyze genetic variation in *INSIG2* in a large school based cohort. By focusing on adolescents from a single school district in Ohio, the heterogeneity which many genetic studies face is minimized. We genotyped 15 single-nucleotide polymorphisms (SNPs): rs7566605, 13 tagging SNPs in *INSIG2*, and rs17047764, located downstream of *INSIG2*. Our hypothesis was that genetic variation in *INSIG2* would be significantly associated with overweight and lipid measures.

## Methods and Procedures

### Population

In this study, we randomly selected 1,058 of the 2,501 students participating in the Princeton School District Study[32], a prospective school-based study of 5^th^ through 12^th^ graders on carbohydrate metabolism, in an urban-suburban school district. The children ranged in age from 10–18 years old and were unrelated. The students we selected (our cohort) had complete data and did not report mixed and/or Hispanic ethnicity. Race and ethnicity was self-reported. Population stratification was not done because this was a candidate gene study and we did not have access to ancestry informative markers. In the Cincinnati area, we have found that self-reported race and ethnicity aligns well with genetic continental ancestry (> 99% concordance). Children were excluded if they had a chronic disease, were taking medication known to affect carbohydrate metabolism, or were pregnant. At the study visit, parents completed a medical history including medication, chronic disease, and history of menarche for girls, and blood was taken by venipuncture after an overnight 10 hour fast. Written informed consent was obtained from all of the parents/guardians, with written assent obtained from all participants. This study was approved by the institutional review boards of Cincinnati Children’s Hospital Medical Center and the University of Cincinnati.

### Anthropometric measures/calculated variables

Height and weight measurements, along with determination of pubertal status, were done with standard procedures and equipment as previously described [32]. BMI was calculated (weight (kg)/height (m)^2^). BMI percentiles for age and sex were determined using the Centers for Disease Control and Prevention growth charts (www.cdc.gov/nccdphp/dnpa/growthcharts/sas.htm). Lean (< 85^th^ BMI %) and overweight (≥85^th^ BMI %) categories of adolescents were defined, consistent with the classification of overweight in children[3].

### Genotyping

We genotyped 15 SNPs: 13 tagging SNPs within *INSIG2*, and SNP rs7566605 (located 10 kb upstream from the transcription start site of *INSIG2)*, and rs17047764 (located downstream from *INSIG2)*. These tagging SNPs were identified using the method of Carlson et al [33] and based on pair-wise r-square (>0.8). These SNPs covered both non-Hispanic white (NHW) and African American (AA) populations.

Blood samples were stored on wet ice immediately after collection, and buffy coats were stored at −80 degrees C until processing. DNA was extracted using Gentra Puregene kits. Genotyping was performed using the SNPlex TM platform (Applied Biosystems), which is based on multiple oligonucleotide ligation/polymerase chain reaction assay with a universal ZipChute TM probe detection from high-throughput multiplexed SNP genotyping. Details of the SNPlex protocol were described previously[34]. To assure genotypic quality, negative controls and blind duplicate samples were introduced in each batch of samples in the 96-well format.

### Statistical Analysis

Analysis was conducted using JMP 7.0. Continuous variables were analyzed for normality. SNPs which deviated from Hardy-Weinberg equilibrium (HWE) were excluded from the analysis. All genetic analyses were conducted in non-Hispanic whites and African Americans separately. Linkage disequilibrium (LD) among SNPs was calculated using r^2^ from JMP. SNP associations assumed an additive effect and were tested using regression (logistic for overweight, and linear for LDL, cholesterol, HDL, and triglycerides). Covariates included age, sex, and puberty stage. Lipid measures were ln transformed and back converted for figures.

To minimize false positive findings due to multiple testing, we accounted for four outcomes: LDL and cholesterol (considered as a single outcome), Overweight, HDL, and Triglycerides. We applied Bonferroni correction to the SNPs which entered the final analysis. Thus for whites, the significance threshold is 0.0013 (0.05/(4 phenotypes * 10 SNPs); for blacks, the significance threshold is 0.001 (0.05/(4 phenotypes * 13 SNPs). However, as we sought to replicate previous reports, nominally significant associations (p ≤ 0.05) were also reported.

## Results

### Characteristics of the Study Population

Participant characteristics are listed in Table 1. Thirty-seven percent were overweight (≥85^th^ BMI %) and sixty-three percent were lean. The African American and non-Hispanic white populations were similar in age, sex, and pubertal status. Of the overweight cohort, forty-four percent were non-Hispanic white.

**Table 1:** Characteristics of the study population.

	**Non-Hispanic White**	**African American**
n	561	497
Age (years)^a^	14.3 ± 2.2	14.2 ± 2.2
Sex (% male)	50	50
Puberty Stage (% pre/peri/post)	12.3/42.1/45.6	11.5/37/51.5
% Overweight (BMI≥85%)	30.1	44.7
BMI (kg/m^2^)^a,b^	22.05 ± 4.91	24.02 ± 6.19
Cholesterol (mg/dl)	151.54 ± 28.76	153.61 ± 27.00
LDL (mg/dl)	88.63 ± 25.27	91.13 ± 23.74
HDL (mg/dl)	45.25 ±10.21	48.80 ± 11.95
Triglycerides (mg/dl)	88.20 ± 42.13	67.88 ± 30.32

^a^Continuous variables presented as means ± s.d.

^b^Significantly different (p < 0.0001).

BMI: Body Mass Index. LDL: low-density lipoprotein. HDL: High-density lipoprotein.

### Genotype exclusions

Genotype calling failed in one SNP, rs10490624, and was excluded from the analysis. In whites, three SNPs, rs13003121, rs2161829, and rs11889497, were not in HWE (p < 0.01), and thus were excluded from the analysis. In African Americans, rs2161829 was not in HWE (p < 0.01), and thus was excluded from analysis. The SNPs included in the analysis, including minor allele frequency (MAF) for both non-Hispanic whites and African Americans, are listed in Tables 2 and 3. The average MAF was 0.22.

**Table 2:** Associations of the variants of the *INSIG2* gene with Overweight and LDL in Non-Hispanic White children.

**SNP**	**Minor Allele**	**MAF**	**Overweight/Obese**		**LDL**		**HDL**		**Triglycerides**	
			p-value	Beta ± se	p-value	Beta ± se	p-value	Beta ± se	p-value	Beta ± se
rs7566605	C	0.31	0.30	−0.02±0.02	0.19	−0.03±0.02	0.82	−0.00±0.02	0.15	−0.04±0.03
rs1352083	T	0.25	0.27	−0.18± 0.16	0.14	0.03±0.02	0.31	−0.02±0.02	0.08	0.06±0.03
rs13393332	C	0.25	0.22	−0.20± 0.16	0.15	0.03±0.02	0.34	−0.02±0.02	0.08	0.06±0.03
rs12464355^b^	G	0.10	0.92	0.02±0.22	2.7 × 10^−5^	−0.12±0.03	0.60	−0.01±0.02	0.46	0.03±0.04
rs2042492	T	0.25	0.31	−0.17±0.16	0.13	0.03±0.02	0.24	−0.02±0.02	0.06	0.06±0.03
rs9808111	A	0.002	—		—		—		—	
rs10490625	T	0.08	0.50	−0.17±0.26	0.76	0.01±0.03	0.52	0.02±0.03	0.66	0.02±0.05
rs889904	A	0.49	0.41	0.11±0.14	0.16	0.03±0.02	0.27	0.02±0.01	0.27	−0.03±0.03
rs17047757^a^	G	0.01	0.043	0.43±0.21	0.39	0.03±0.03	0.58	0.01±0.02	0.89	−0.01±0.05
rs11889497^c^	G	0.07	—		—		—		—	
rs9308762	C	0.17	0.41	0.14±0.17	0.72	0.01±0.02	0.73	0.01±0.02	0.37	−0.03±0.04
rs13003121^c^	A	0.04	—		—		—		—	
rs17047764	C	0.16	0.49	−0.13±0.19	0.79	0.06±0.02	0.55	−0.01±0.02	0.41	0.03±0.04

^a^Associated with overweight (BMI ≥ 85% for age) in Non-Hispanic Whites.

^b^Associated with LDL in Non-Hispanic Whites.

^c^Out of Hardy-Weinberg equilibrium in whites

SNP: Single nucleotide polymorphism. MAF: minor allele frequency. *INSIG2*: Insulin-induced gene 2. LDL: low-density lipoproteins. HDL: high-density lipoproteins. SE: standard error.

Associations were made after adjusting for age, sex, and puberty stage using regression. To adjust for multiple testing, a p-value of 0.0013 was considered statistically significant, Bonferroni correction of (0.05/(4 phenotypes *10 SNPS).

**Table 3:** Associations of the variants of the *INSIG2* gene with Overweight and LDL in African-American children.

**SNP**	**Minor Allele**	**MAF**	**Overweight/Obese**		**LDL**		**HDL**		**Triglycerides**	
			**p-value**	**Beta ± se**	**p-value**	**Beta ± se**	**p-value**	**Beta ± se**	**p-value**	**Beta ± se**
rs7566605	C	0.26	0.84	−0.03±0.02	0.89	0.00±0.02	0.82	0.00±0.02	0.29	−0.03±0.03
rs1352083	T	0.27	0.45	−0.11±0.15	0.05	0.04±0.19	0.97	−0.00±0.02	0.45	0.02±0.03
rs13393332	C	0.27	0.49	−0.10±0.14	0.04	0.04±0.02	0.87	0.00±0.02	0.42	0.02±0.03
rs12464355	G	0.02	0.87	0.09±0.53	0.79	−0.02±0.07	0.93	0.00±0.07	0.34	0.10±0.10
rs2042492	T	0.27	0.48	−0.10±0.15	0.04	0.04±0.02	0.96	0.00±0.02	0.47	0.02±0.03
rs9808111	A	0.06	0.57	0.15±0.27	0.27	0.04±0.04	0.21	0.04±0.03	0.63	−0.03±0.05
rs10490625	T	0.02	0.18	0.66±0.49	0.26	0.07±0.07	0.97	0.00±0.06	0.22	0.11±0.10
rs889904	A	0.58	0.27	−0.15±0.14	0.17	−0.03±0.02	0.84	−0.00±0.02	0.014	−0.06±0.03
rs17047757	G	0.03	0.51	−0.27±0.40	0.97	−0.00±0.05	0.93	−0.00±0.05	0.59	−0.04±0.08
rs11889497	G	0.24	0.61	0.08±0.15	0.39	0.02±0.02	0.32	0.02±0.02	0.23	0.03±0.03
rs9308762	C	0.17	0.92	−0.02±0.18	0.07	−0.05±0.03	0.12	−0.04±0.02	0.39	−0.03±0.04
rs13003121^c^	A	0.09	0.13	−0.37±0.24	0.72	−0.01±0.03	0.25	0.03±0.03	0.74	0.02±0.04
rs17047764	C	0.38	0.45	0.10±0.14	0.67	−0.01±0.02	0.67	0.01±0.02	0.12	0.04±0.03

SNP: Single nucleotide polymorphism. MAF: minor allele frequency. *INSIG2*: Insulin-induced gene 2. LDL: low-density lipoproteins. HDL: high-density lipoproteins. SE: Standard error.

Associations were made after adjusting for age, sex, and puberty stage using regression. To adjust for multiple testing, a p-value of 0.001 was considered statistically significant, Bonferroni correction of (0.05/(4 phenotypes * 13 SNPs).

### SNP of *INSIG2* associated with overweight

**Figure 1:**
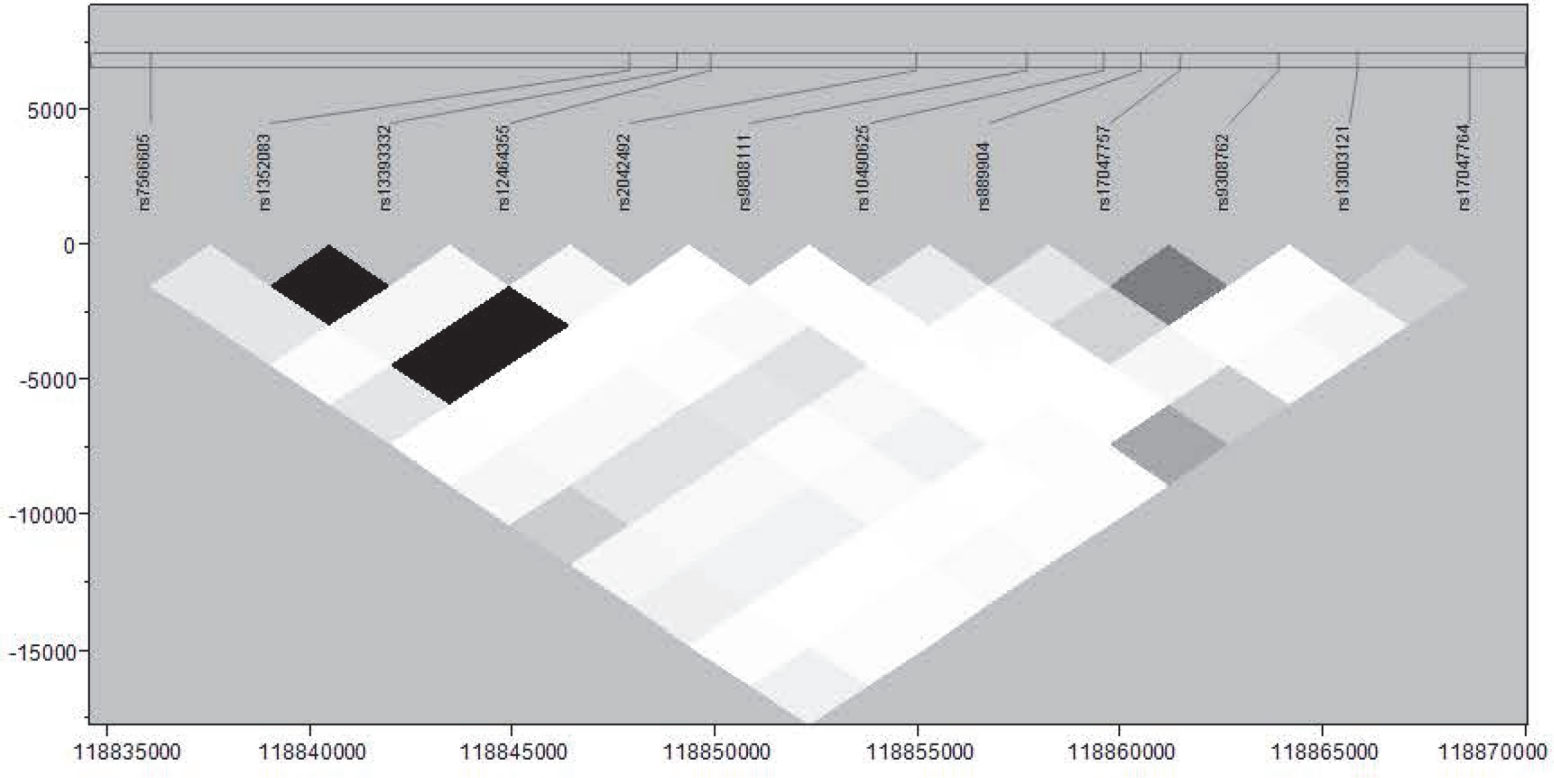
Linkage disequilibrium as measured by r^2^ across *INSIG2* in Non-Hispanic Whites.

**Figure 2:**
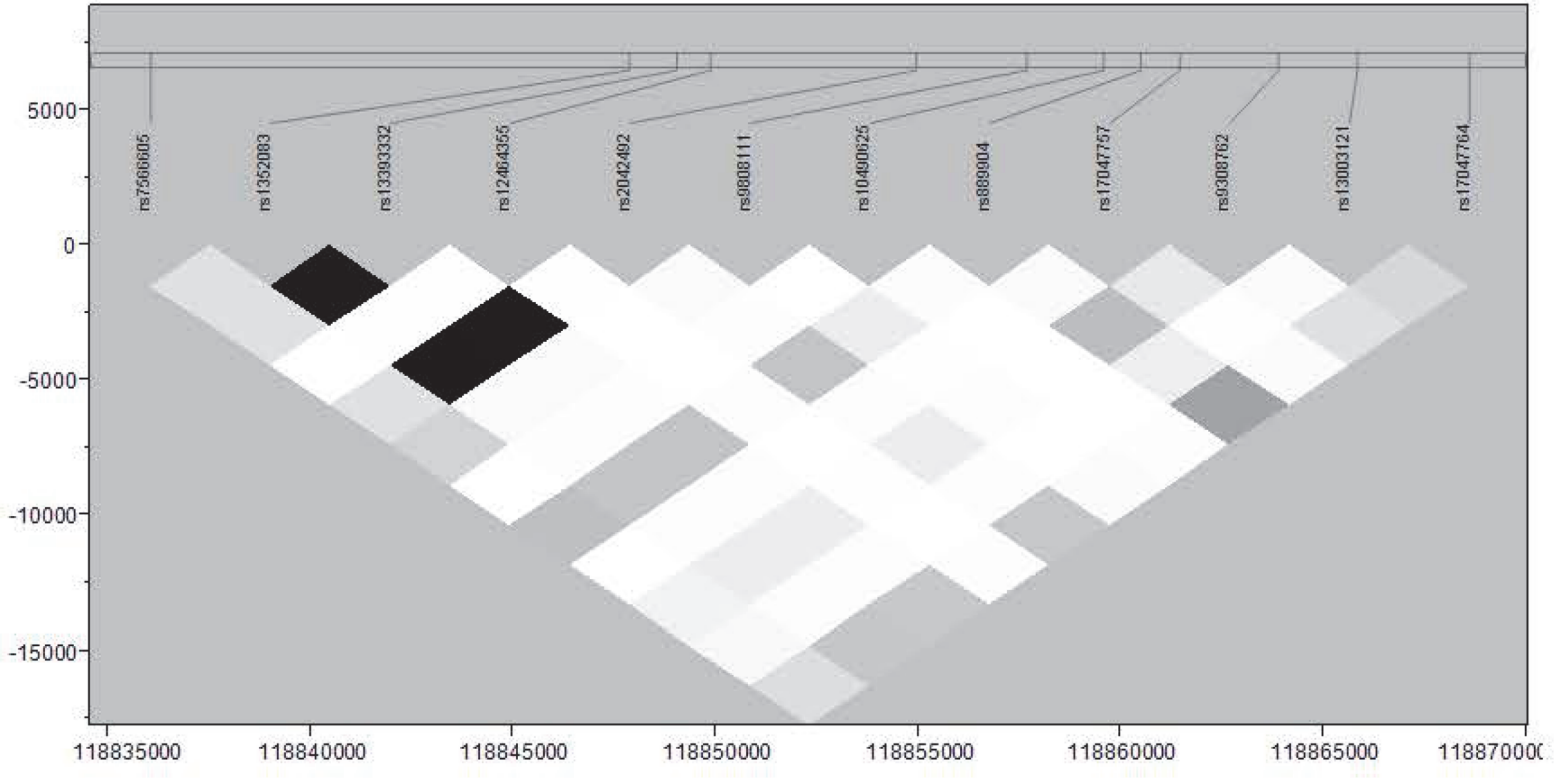
Linkage disequilibrium as measured by r^2^ across *INSIG2* in African-Americans.

Results of association testing between *INSIG2* SNPs and overweight (p-values and beta estimates) are presented in Tables 2 and 3 for whites and blacks respectively. We did not find an association between overweight and rs7566605, but we did find a nominally significant association with overweight and rs17047757 in NHW children (p = 0.043). The G allele of rs17047757 has an odds ratio of 1.5 for overweight in children, (95% confidence interval 1.01–2.32). This SNP is in LD with rs7566605, r^2^ of 0.20. LD plot for whites is shown in Fig. 1. No significant associations were identified in the African American cohort (Table 3) even though the LD structure (Fig. 2) was markedly similar to whites.

### SNP of *INSIG2* associated with LDL

**Figure 3:**
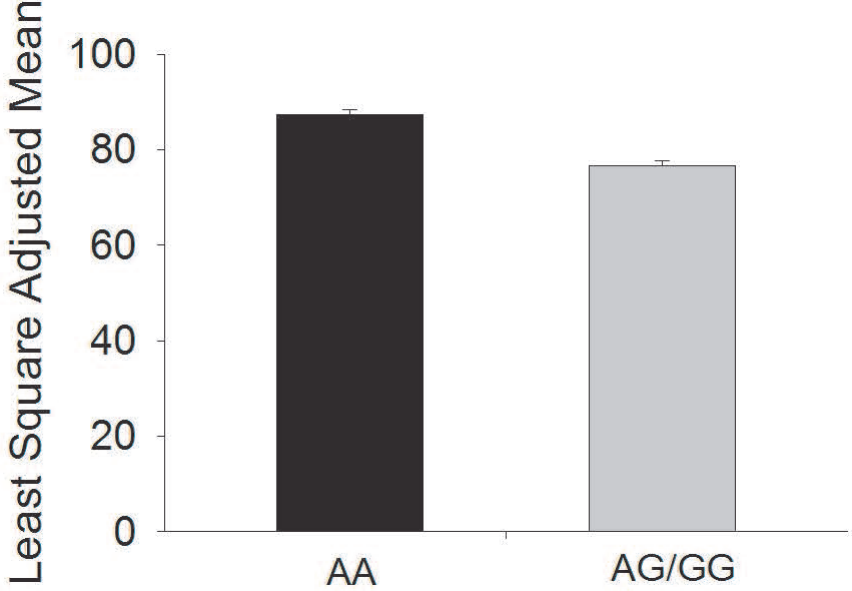
G allele of SNP rs12464355 in the *INSIG2* gene is associated with lower LDL levels, p< 0.001 in Non-Hispanic Whites. Least Square Adjusted Mean of LDL (mg/dl) is shown. Predicted values are given from a multiple regression analysis, adjusting for age, gender, and pubertal stage. LDL levels were *ln* transformed and back converted.

We also found a strong significant association between rs12464355 and LDL in NHW children, p < 0.001 (Table 2). The minor allele, G, is protective, as shown in Fig. 3. Patients with AG or GG had lower LDL levels. We found no association with rs7566605 and cholesterol, LDL, triglycerides, or HDL (p > 0.15). In African Americans, no significant associations were detected (Table 3).

## Discussion

In this study, we confirmed that genetic variation in *INSIG2* is associated with both overweight and LDL in NHW children. Although we failed to find these associations with the SNP rs7566605, we identified a novel genetic association between SNP rs17047757 and overweight. In addition, we identified a novel, protective genetic association between SNP rs12464355 and LDL. These data support the concept that polymorphisms in *INSIG2* appear to be important in the development of obesity through its effects on lipid regulation, but perhaps not through the previously associated variant.

Previous studies have found possible associations between *INSIG2* polymorphisms and lipid synthesis. Tiwari et al observed a non-significant trend in the C allele of rs7566605 and antipsychotic medication induced weight gain. Similarly, Le Hellard et al reported a strong association of three SNPs located within or near the *INSIG2* gene (rs17587100, rs10490624, and rs17047764) with antipsychotic medication-related weight gain. In contrast, Oki et al reported a positive association between the C allele of SNP rs7566605 and lower cholesterol in Japanese-American females, which suggests that this SNP may be protective when exposed to a high fat diet [22].

Our study also found a protective effect on LDL (those with the minor allele, G, of SNP rs12464355 had lower levels). This finding replicates a previous report of an association between rs12464355 and LDL[35]). Our failure to identify association between SNP rs7566605 and lipid levels is consistent with previous studies [12,13,14,24,26,27].

We also identified a polymorphism of the *INSIG2* gene with overweight in children, but it is a different SNP than those identified by previous studies. Herbert et al[4] published the first study to implicate the C allele of the SNP rs7566605 in association with BMI. They found this strong association in a large genome-wide association study, and replicated the findings in 4 other separate populations. Since then, several other studies have also found a significant association with rs7566605 and obesity or BMI[9,10,11,12], but some did not[8,13–29], including this study.

The discordance between our data and previous studies suggest that another genetic variation of *INSIG2* may be important in adiposity. The SNP rs7566605 may not be the causative SNP for adiposity, but may be in linkage disequilibrium with the causative SNP. Our study is one of the first to look at other tagging SNPs, and our novel association is in LD with rs7566605.

Other studies support this idea that the causative SNP may be in LD with rs7566605. Krapivner et al[8] found that the G allele of the −102G/A promoter in *INSIG2* is significantly associated with BMI, and may be the functional polymorphism. Ciullo et al [15] did not find an association between rs7566605 and BMI or obesity, but they detected a locus on chromosome 2q14.3 that displayed linkage with BMI and obesity in both populations. This locus contains the rs7566605 SNP.

A limitation to our study is that, although our sample size was large, it may not have been large enough to detect an influence of rs7566605 on overweight in children. However, our population is unique in that we were able to study children from one geographic region, who were all in the same school district, which reduces the degree of heterogeneity. One variable that limits our ability to compare our study to others is the variability in phenotypic definition. Some studies looked at overweight versus lean, and others did not.

## Conclusion

We identified a novel SNP in the *INSIG2* gene that is associated with overweight in NHW children, rs17047757, and one SNP that is associated with LDL in NHW children, rs12464355. We did not find an association with overweight or lipid profiles in children and rs7566605. These data suggest that polymorphisms in *INSIG2* may be important in the development of obesity through its effects on lipid regulation.
